# Superior Mesenteric Artery Syndrome Presenting With Refractory Vomiting and Acute Kidney Injury: A Case Report

**DOI:** 10.7759/cureus.108101

**Published:** 2026-05-01

**Authors:** Fatma Elsayed, Omar Mahrous, Mohamed Abdulmajeed, Ahmed Elsebahy, George Macfaul

**Affiliations:** 1 Gastroenterology, Milton Keynes University Hospital, Milton Keynes, GBR; 2 General Internal Medicine, Ysbyty Gwynedd Hospital, Bangor, GBR; 3 General Internal Medicine, Milton Keynes University Hospital, Milton Keynes, GBR; 4 Radiology, Milton Keynes University Hospital, Milton Keynes, GBR

**Keywords:** aortomesenteric angle, computed tomography abdomen, gastrointestinal obstruction, persistent vomiting, superior mesenteric artery syndrome

## Abstract

Superior mesenteric artery syndrome (SMAS) is a rare cause of upper gastrointestinal obstruction caused by compression of the third portion of the duodenum between the abdominal aorta and the superior mesenteric artery, usually due to rapid weight loss or an anatomical predisposition that reduces the retroperitoneal fat pad between these vessels. Its symptoms often mimic more common gastrointestinal disorders, which can delay diagnosis, making cross-sectional imaging, particularly computed tomography (CT), essential for identifying a reduced aortomesenteric angle and distance with proximal duodenal dilatation. We report the case of a 17-year-old female patient who presented with persistent nausea, vomiting, and inability to tolerate oral intake despite extensive gastroenterological evaluation; upper gastrointestinal endoscopy and magnetic resonance cholangiopancreatography were unremarkable, but CT subsequently demonstrated an aortomesenteric angle of approximately 17° and a distance of approximately 7 mm with proximal duodenal dilatation, consistent with SMAS. She was managed conservatively with nutritional optimisation and supportive therapy. This case highlights the diagnostic challenge of SMAS and underscores the importance of considering it in patients with unexplained persistent vomiting, as early recognition and appropriate imaging are crucial to prevent prolonged morbidity and unnecessary investigations.

## Introduction

Superior mesenteric artery syndrome (SMAS), also known as Wilkie’s syndrome, is a rare but clinically significant cause of upper gastrointestinal obstruction resulting from compression of the third portion of the duodenum between the abdominal aorta and the superior mesenteric artery [1]. Despite being described over a century ago, it remains an underdiagnosed condition due to its nonspecific clinical presentation and overlap with more common gastrointestinal disorders [2].

Under normal anatomical conditions, the superior mesenteric artery arises from the anterior surface of the abdominal aorta at approximately the level of the first lumbar vertebra (L1) and forms an angle ranging between 25° and 60° with the aorta [3]. The third part of the duodenum passes through this space, which is normally maintained by a cushion of mesenteric fat and lymphatic tissue. This fat pad plays a critical role in preventing vascular compression of the duodenum. However, when this protective layer is reduced, the aortomesenteric angle narrows, and the aortomesenteric distance decreases, leading to compression of the duodenum and subsequent obstruction [4].

Radiologically, SMAS is defined by an aortomesenteric angle of less than 22° and an aortomesenteric distance of less than 8 mm. These parameters are most accurately measured using contrast-enhanced computed tomography (CT), which also demonstrates associated features such as dilatation of the stomach and proximal duodenum with abrupt narrowing at the level of vascular compression [4].

The prevalence of SMAS is estimated to range between 0.013% and 0.3%, reflecting its rarity in clinical practice [5]. It most commonly affects adolescents and young adults, with a higher prevalence reported in women. Several predisposing factors have been identified, including rapid weight loss, eating disorders such as anorexia nervosa, chronic illness, malignancy, trauma, burns, and postoperative states, particularly following scoliosis correction [6].

Clinically, SMAS presents with nonspecific gastrointestinal symptoms including postprandial epigastric pain, early satiety, nausea, and recurrent vomiting. These symptoms are often exacerbated after meals and may lead to further weight loss, thereby worsening the anatomical compression and creating a vicious cycle [7]. In some cases, symptoms may improve with positional changes such as lying in the left lateral decubitus or knee-chest position, which transiently increases the aortomesenteric angle.

Due to the nonspecific nature of its presentation, SMAS is frequently misdiagnosed or diagnosed late, with patients often undergoing multiple investigations before the correct diagnosis is established. Cross-sectional imaging, particularly CT angiography, plays a crucial role in confirming the diagnosis by demonstrating reduced aortomesenteric angle and distance, along with proximal duodenal dilatation [8].

Management strategies for SMAS depend on the severity of symptoms and duration of illness. Conservative treatment, including nutritional rehabilitation and weight restoration, is considered first-line therapy and aims to increase retroperitoneal fat and widen the aortomesenteric angle. Surgical intervention, most commonly duodenojejunostomy, is reserved for patients who fail to respond to conservative measures [9,10].

## Case presentation

A 17-year-old female patient presented with persistent nausea, abdominal discomfort, and recurrent vomiting that had progressively worsened over several weeks. She reported an inability to tolerate oral intake and described episodes of retching shortly after attempting to eat. The symptoms were associated with epigastric discomfort but no fever, gastrointestinal bleeding, or changes in bowel habit.

Her medical history included functional dyspepsia and iron deficiency anaemia. She was under outpatient follow-up with gastroenterology services. Her regular medications included nortriptyline 25 mg once daily and omeprazole 20 mg daily. She denied alcohol consumption, smoking, or illicit drug use. She was currently attending a sixth form college and lived with her family.

On admission, the patient’s weight was 38.5 kg. BMI and prior weight records were not available. She reported reduced oral intake over the preceding weeks due to persistent nausea and vomiting.

On examination, the patient appeared uncomfortable but haemodynamically stable. Vital signs were within normal limits. Abdominal examination revealed mild epigastric tenderness without guarding or rebound tenderness. Bowel sounds were present, and no palpable masses were identified.

Initial laboratory investigations demonstrated significant abnormalities consistent with severe dehydration and physiological stress, including haemoconcentration, marked leucocytosis, hypokalaemia, acute kidney injury, and elevated lactate (Table 1). These abnormalities improved with intravenous fluid resuscitation and supportive management.

**Table 1:** Laboratory findings on admission and during hospital stay. The elevated haemoglobin on admission was attributed to haemoconcentration secondary to severe dehydration. The subsequent haemoglobin value of 107 g/L after fluid resuscitation was considered more reflective of the patient’s underlying iron deficiency anaemia. AKI: acute kidney injury; eGFR: estimated glomerular filtration rate

Parameter	Admission	During admission	Reference range	Interpretation
Haemoglobin	171 g/L	107 g/L	120-160 g/L	Haemoconcentration → dilution after fluids
White cell count	26.54	15.23	4-11 x 10⁹/L	Stress response
Platelets	321	148	150-400 x 10⁹/L	Normal trend
Potassium	2.6 mmol/L	2.9 mmol/L	3.5-5.0 mmol/L	Hypokalaemia (vomiting)
Urea	33.2 mmol/L	12.5 mmol/L	2.5-7.8 mmol/L	Severe dehydration
Creatinine	334 µmol/L	149 µmol/L	45-90 µmol/L	AKI improving
eGFR	16	42	>60	AKI stage 3 improving
CRP	7.4 mg/L	16 mg/L	<5 mg/L	Mild inflammation
Magnesium	1.29	-	0.7-1.0 mmol/L	Elevated
Phosphate	2.76	-	0.8-1.5 mmol/L	Elevated
Lactate	6.4 → 5.8	-	<2 mmol/L	Hypoperfusion

Upper gastrointestinal endoscopy was performed and demonstrated normal mucosa throughout the oesophagus, stomach, and duodenum. No structural abnormalities were detected. *Helicobacter pylori* testing was negative.

**Figure 1:**
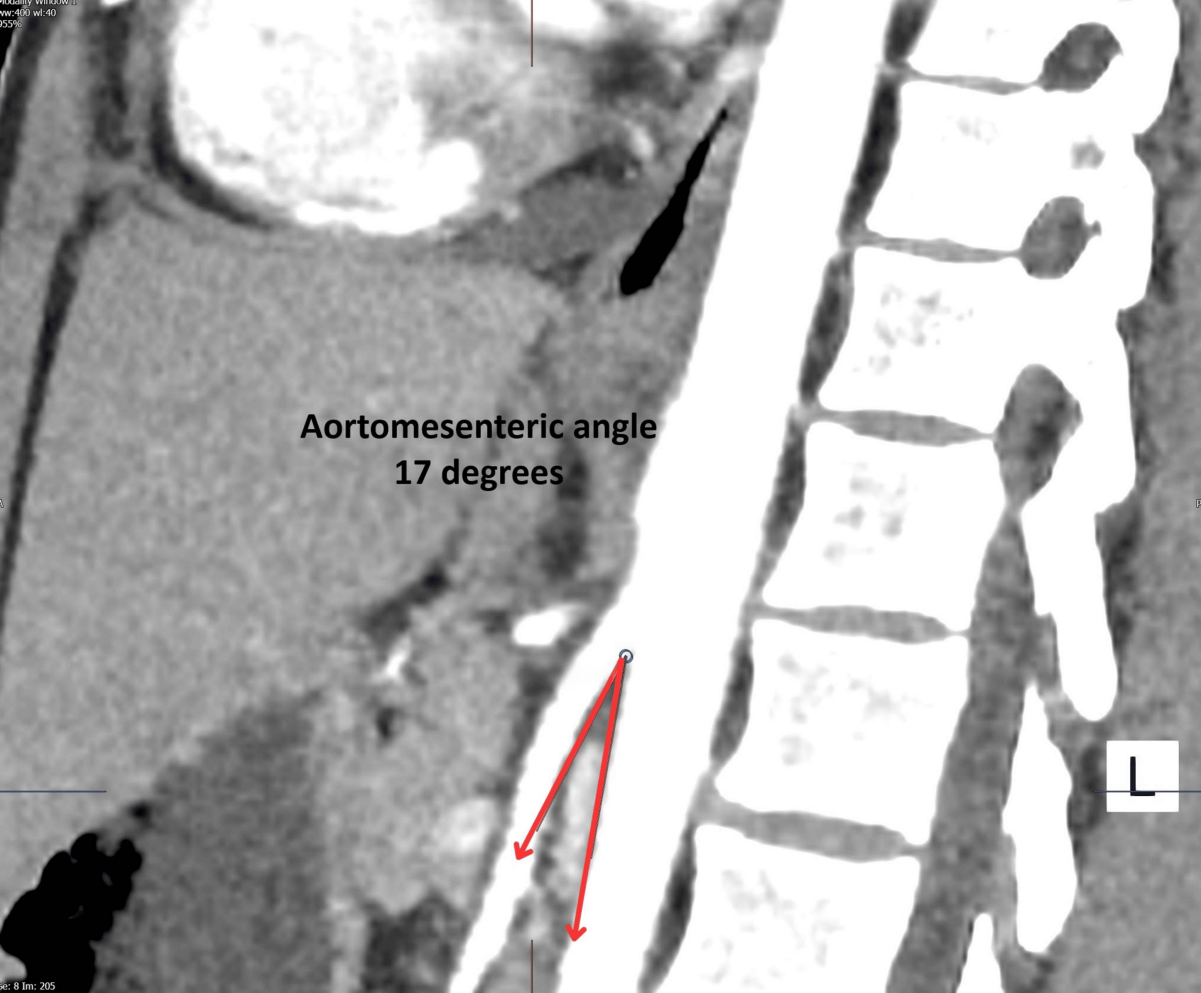
Sagittal contrast-enhanced CT image demonstrating a reduced aortomesenteric angle (17°) between the abdominal aorta and superior mesenteric artery, consistent with superior mesenteric artery syndrome. CT: computed tomography

**Figure 2:**
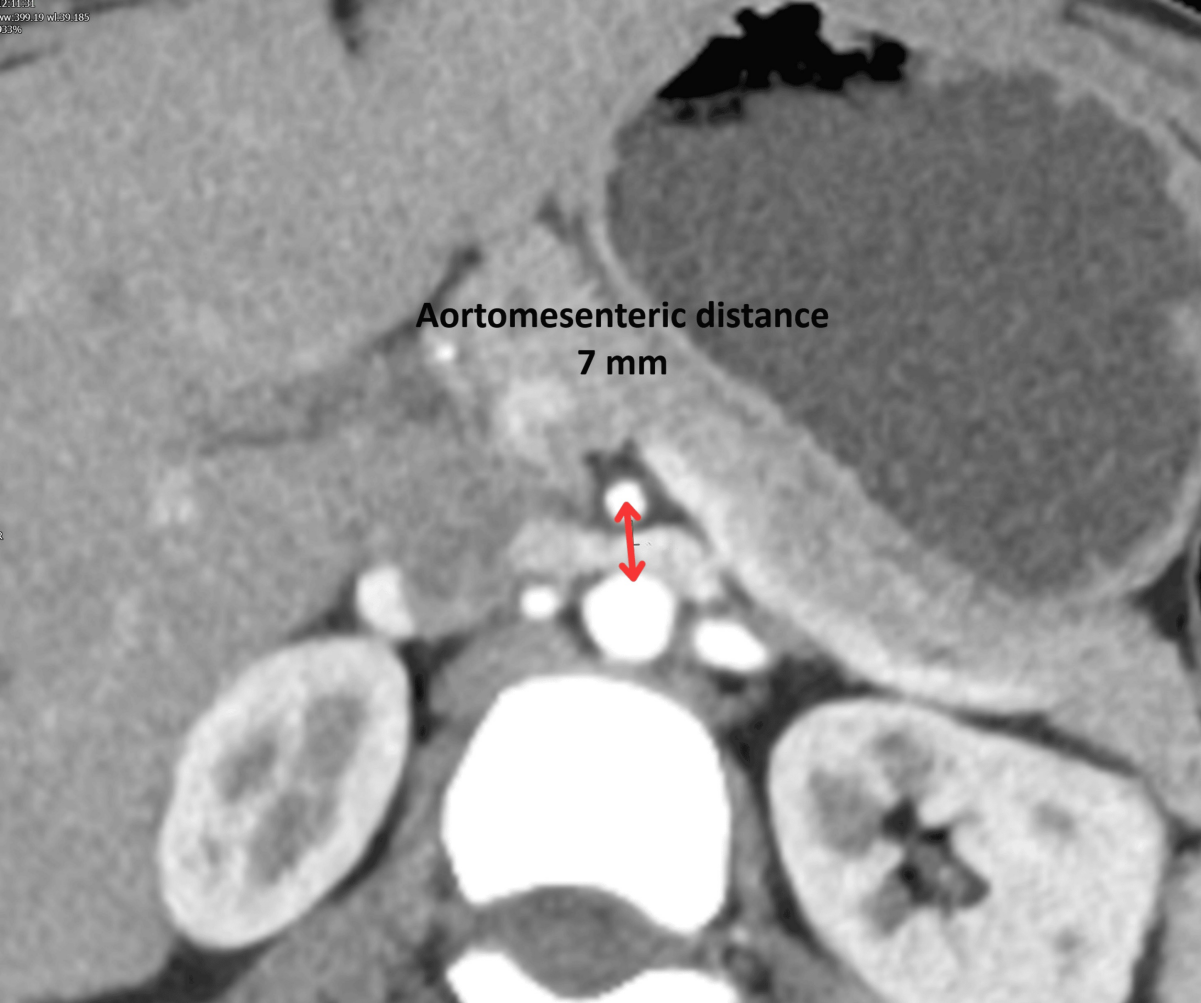
Axial contrast-enhanced CT image demonstrating reduced aortomesenteric distance (~7 mm) with compression of the third part of the duodenum between the aorta and superior mesenteric artery. CT: computed tomography

Magnetic resonance cholangiopancreatography was subsequently performed to exclude biliary or pancreatic pathology and revealed no abnormalities. Despite these investigations, the patient continued to experience persistent vomiting and an inability to tolerate oral intake. A contrast-enhanced CT scan of the abdomen was therefore performed (Figures 1, 2).

CT imaging demonstrated marked gastric and proximal duodenal dilatation with narrowing of the third part of the duodenum at the level of the superior mesenteric artery. Measurement of the vascular anatomy revealed narrowing of the aortomesenteric angle to approximately 17° with an aortomesenteric distance of approximately 7 mm (Table 2).

**Table 2:** Radiological criteria for diagnosis of SMAS. SMAS: superior mesenteric artery syndrome

Parameter	Normal range	SMAS diagnostic range	Patient value
Aortomesenteric angle	25°-60°	<22°	17°
Aortomesenteric distance	10-28 mm	<8 mm	~7 mm
Duodenal compression	Absent	Present	Present
Gastric dilatation	Absent	Present	Present

These findings were consistent with compression of the duodenum between the abdominal aorta and the superior mesenteric artery, supporting the diagnosis of SMAS. The patient was managed conservatively with aggressive intravenous fluid resuscitation over the initial 48-72 hours to correct dehydration and acute kidney injury, alongside careful electrolyte replacement, particularly for hypokalaemia. Antiemetic therapy, including intravenous ondansetron and metoclopramide as required, was administered to control persistent nausea and vomiting. Over the first few days of admission, there was a gradual improvement in vomiting frequency and biochemical parameters. Oral intake was slowly reintroduced and progressively tolerated.

A structured nutritional rehabilitation plan was initiated early during admission, with a focus on gradual reintroduction of oral intake. The patient was encouraged to adopt small, frequent meals with a preference for high-calorie, easily digestible foods. Nutritional supplementation was provided where tolerated, and dietetic input was sought to optimise caloric intake and minimise further weight loss. Postural measures, including maintaining a left lateral or semi-recumbent position after meals, were also advised to reduce duodenal compression and improve symptom tolerance.

Over the course of admission, the patient demonstrated gradual clinical improvement, with a reduction in vomiting frequency, improved oral intake, and stabilisation of renal function and electrolyte levels. Her symptoms became manageable with conservative measures alone, and no surgical intervention was required during this admission.

The patient was discharged after three days of hospitalisation once clinically stable, with a clear plan for ongoing nutritional optimisation and symptom monitoring. She was arranged for close outpatient follow-up under the gastroenterology team, with further review of nutritional status, symptom progression, and consideration of additional interventions should conservative management fail.

## Discussion

SMAS is an uncommon but clinically important cause of proximal intestinal obstruction resulting from external compression of the third portion of the duodenum between the abdominal aorta and the superior mesenteric artery [1]. Despite being recognised for many years, it remains an underdiagnosed condition due to its rarity and the nonspecific nature of its presenting symptoms, which frequently mimic more common gastrointestinal disorders [2].

The pathophysiology of SMAS is primarily related to alterations in the anatomical relationship between the abdominal aorta and the superior mesenteric artery. In normal individuals, the aortomesenteric angle typically ranges between 25° and 60°, whilst the aortomesenteric distance ranges between 10 and 28 mm, allowing sufficient space for the third portion of the duodenum to pass without compression [3]. A reduction in retroperitoneal fat, which normally acts as a cushion between these vascular structures, results in narrowing of the angle and distance, ultimately leading to compression of the duodenum and functional obstruction [4].

Several predisposing factors have been described, with rapid weight loss being the most significant and commonly reported. This may occur in patients with eating disorders, malignancy, chronic debilitating illness, trauma, burns, or prolonged immobilisation [5]. In addition, postoperative states, particularly following spinal surgery or scoliosis correction, have been associated with acute changes in the aortomesenteric angle. In the present case, the patient had a low admission weight of 38.5 kg and a history of reduced oral intake due to persistent nausea and vomiting. Although objective weight trajectory data were not available, these factors suggest a low body mass state, which may have contributed to the depletion of mesenteric fat and predisposed to the development of SMAS.

The clinical presentation of SMAS is often insidious and nonspecific. Patients commonly report postprandial epigastric pain, early satiety, nausea, and recurrent vomiting [6]. These symptoms are frequently exacerbated after meals and may improve with positional changes such as the knee-chest or left lateral decubitus position, which transiently relieve vascular compression. The nonspecific nature of these symptoms often results in misdiagnosis as more common conditions such as gastritis, functional dyspepsia, or gastroparesis, leading to delays in diagnosis and appropriate management [7].

Radiological imaging is central to the diagnosis of SMAS, with contrast-enhanced CT considered the gold standard. CT imaging allows direct visualisation of the vascular anatomy and accurate measurement of the aortomesenteric angle and distance. Typical findings include gastric and proximal duodenal dilatation, abrupt narrowing of the third part of the duodenum, and reduced aortomesenteric angle and distance [8]. In this patient, CT demonstrated an aortomesenteric angle of 17° and a distance of approximately 7 mm, which fall well within the diagnostic criteria for SMAS and strongly support the diagnosis. These findings correlate with previous studies demonstrating the reliability of CT in identifying vascular compression and guiding clinical management [9].

Management of SMAS is primarily dependent on the severity and chronicity of symptoms. Initial treatment is typically conservative and focuses on nutritional rehabilitation, which aims to restore retroperitoneal fat and increase the aortomesenteric angle [9]. This approach may involve oral nutritional supplementation, enteral feeding via nasojejunal tubes, or, in more severe cases, parenteral nutrition. Adjunctive measures such as postural therapy may also provide symptomatic relief by temporarily reducing duodenal compression.

Surgical intervention is considered in patients who fail to respond to conservative management or who develop persistent or severe symptoms. The most commonly performed surgical procedure is duodenojejunostomy, which bypasses the compressed segment of the duodenum and has been shown to provide excellent long-term outcomes [10]. Alternative procedures, such as Strong’s procedure or gastrojejunostomy, may be considered in selected cases but are less frequently utilised.

This case illustrates several important clinical considerations. Firstly, SMAS should be included in the differential diagnosis of patients presenting with persistent unexplained vomiting, particularly in the presence of weight loss or malnutrition. Secondly, early utilisation of cross-sectional imaging is essential for establishing the diagnosis and avoiding unnecessary investigations. Finally, timely recognition and appropriate management are crucial in preventing complications such as severe dehydration, electrolyte imbalance, and acute kidney injury, all of which were evident in this patient.

Similar cases reported in the literature emphasise the importance of maintaining a high index of suspicion and early imaging. For example, Shen et al. described a case of SMAS presenting with persistent vomiting and weight loss, where diagnosis was confirmed by CT demonstrating a reduced aortomesenteric angle and distance [10]. This is comparable to our case in terms of clinical presentation and radiological findings; however, our patient’s presentation was further complicated by severe dehydration and acute kidney injury, highlighting the potential for significant metabolic derangement when diagnosis is delayed. Overall, this case reinforces the need for increased awareness of SMAS among clinicians and highlights the importance of integrating clinical findings with radiological evidence to achieve timely diagnosis and effective management.

## Conclusions

SMAS is a rare but important cause of upper gastrointestinal obstruction that is often underdiagnosed because its symptoms, including nausea, vomiting, early satiety, and weight loss, overlap with more common gastrointestinal conditions. This case highlights the need to maintain a high index of suspicion for SMAS in patients with persistent unexplained vomiting, particularly in the setting of weight loss or malnutrition, and reinforces the value of contrast-enhanced CT in demonstrating a reduced aortomesenteric angle and distance with duodenal compression. Early diagnosis and conservative management are essential, particularly in severe presentations such as this case, which was complicated by significant dehydration and acute kidney injury, to prevent adverse outcomes.
